# Rhodium-catalyzed intramolecular reductive aldol-type cyclization: Application for the synthesis of a chiral necic acid lactone

**DOI:** 10.3762/bjoc.18.176

**Published:** 2022-12-02

**Authors:** Motoyuki Isoda, Kazuyuki Sato, Kenta Kameda, Kana Wakabayashi, Ryota Sato, Hideki Minami, Yukiko Karuo, Atsushi Tarui, Kentaro Kawai, Masaaki Omote

**Affiliations:** 1 School of Pharmacy at Fukuoka, International University of Health and Welfare, 137-1 Enokizu, Okawa, Fukuoka 831-8501, Japan; 2 Faculty of Pharmaceutical Sciences, Setsunan University, 45-1, Nagaotoge-cho, Hirakata, Osaka 573-0101, Japan; 3 Faculty of Pharmaceutical Sciences, Hiroshima International University, 5-1-1 Hirokoshingai, Kure, Hiroshima 737-0112, Japan

**Keywords:** β-hydroxylactone, intramolecular reductive aldol cyclization, necic acid lactone, rhodium catalyst

## Abstract

A rhodium-catalyzed intramolecular reductive aldol-type cyclization is described to give β-hydroxylactones with high diastereoselectivities. The stereoselectivity of this cyclization is highly solvent dependent and can give *syn*- or *anti*-β-hydroxylactones with high diastereoselectivity. This methodology was also applied to the synthesis of a chiral necic acid lactone which is a structural component of the pyrrolizidine alkaloid monocrotaline.

## Introduction

**Scheme 1:**
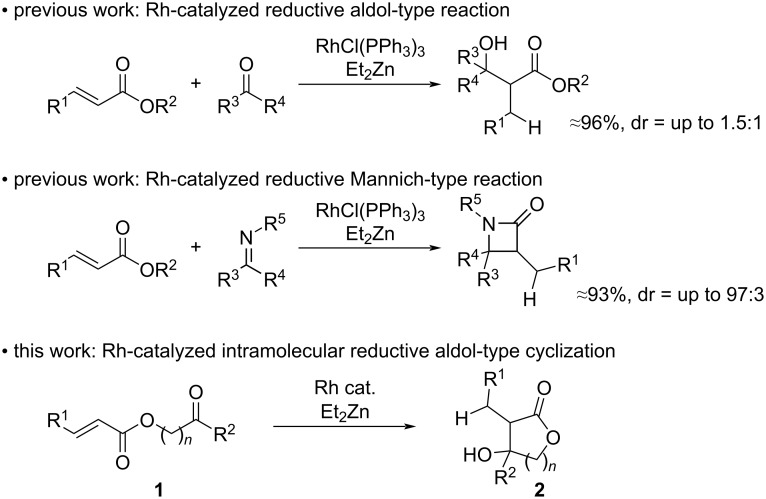
Previous works and this work.

Carbon–carbon bond-forming reactions are among the most important reactions in the synthetic chemistry toolbox and the aldol reaction is one of the most powerful tools to achieve this transformation [1–8]. In particular, the intramolecular aldol condensation is an important approach to the formation of ring systems such as cyclic β-hydroxy carbonyl products or cyclic α,β-unsaturated carbonyl products. Therefore, various types of intramolecular aldol-type reactions have been developed and widely applied to the total synthesis of diverse natural products [9–18]. The reductive aldol-type reaction is another important variation that has been reported using metal catalysts such as Co [19–21], Cu [22–25], and others [26–32] with hydrosilanes (R_3_Si-H) or hydrogen as the reductant. In this area, rhodium catalysis has received significant attention [33–40], and we have also reported reductive α-acylations, reductive aldol-type reactions, and reductive Mannich-type reactions using RhCl(PPh_3_)_3_ with Et_2_Zn [41–47]. The rhodium-catalyzed reductive aldol reaction of α,β-unsaturated esters with aldehydes or ketones gives aldol-type products in good to excellent yields (Scheme 1) [43–44]. In addition, the reductive aldol-type reaction could also be applied to an asymmetric system, although the diastereoselectivity was poor. On the other hand, reductive Mannich-type reactions were achieved in good to excellent yields with high diastereoselectivity [45–46]. As part of a wider program of C–C bond formation systems, we herein report a rhodium-catalyzed intramolecular reductive aldol-type cyclization and its application for the synthesis of a chiral necic acid lactone.

## Results and Discussion

### Rh-catalyzed intramolecular cyclization

When applying our previously reported conditions [43], the intramolecular reductive aldol-type cyclization of **1a** proceeded smoothly and gave the desired product **2a** in a good yield, but the diastereomeric ratio was not sufficient as shown in entry 1 (Table 1). To improve the diastereoselectivity of the reaction, we optimized the conditions for the reductive aldol-type reaction by intramolecular cyclization of **1**, and the results are summarized in Table 1. The use of [RhCl(cod)]_2_ in dichloromethane gave the best result with high diastereoselectivity, and the stereochemistry of the major product **2a** was found to be the *syn*-form with regard to the CH_3_ (C^α^) and OH (C^β^) moieties (Table 1, entry 7). Interestingly, using the higher coordinating solvents, DMF or DMPU, preferentially gave the opposite diastereomer, i.e., the major product being the *anti*-form with regard to CH_3_ (C^α^) and OH (C^β^) moieties (*anti*-**2a**, Table 1, entries 10 and 11). For stereochemistry assignment, the relative configurations of *syn*-**2a** and *anti*-**2a** were confirmed by X-ray crystallography. In addition, a NOESY experiment of the product *syn*-**2a** showed an nOe correlation between the methine proton on C^α^ and one of the protons of the benzene ring on C^β^, but not in *anti*-**2a**.

**Table 1:** Optimization of the reaction conditions.

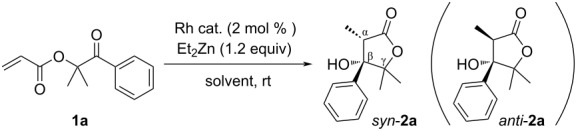					

Entry	Rh cat.	Solvent	Time (h)	Yield^a^	dr [*syn*:*anti*]^b,c^

1	RhCl(PPh_3_)_3_	THF	1	64	[3:1]
2	[RhCl(cod)]_2_	THF	1.5	72	[30:1]
3	RhClCO(PPh_3_)_2_	THF	1	85	[9:1]
4	Rh(acac)(CO)_2_	THF	1	trace	–
5	[RhCl(cod)]_2_	toluene	1	68	[14:1]
6	[RhCl(cod)]_2_	AcOEt	1	79	[25:1]
7	[RhCl(cod)]_2_	CH_2_Cl_2_	1	77	[31:1]
8	[RhCl(cod)]_2_	DME	2	17^d^	–
9	[RhCl(cod)]_2_	CH_3_CN	1	71	[2:1]
10	[RhCl(cod)]_2_	DMF	3	42	[1:27]
11	[RhCl(cod)]_2_	DMPU	1	52	[1:50]

^a^Isolated yield; ^b^the stereochemistry between CH_3_ (C^α^) and OH (C^β^) moieties; ^c^diastereomeric ratio was determined after purification; ^d^diastereomeric mixture.

**Scheme 2:**
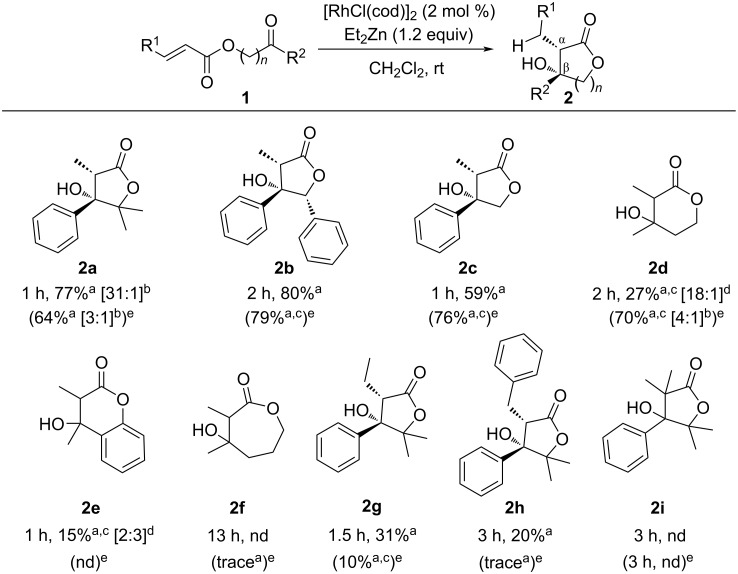
Scope and limitation of the rhodium-catalyzed reductive aldol-type cyclization. ^a^Isolated yield. ^b^Diastereomeric ratio was determined after purification. ^c^Diastereomeric mixture. ^d^Diastereomeric ratio was determined by ^1^H NMR. ^e^The reaction was carried out using RhCl(PPh_3_)_3_ in THF at rt.

Next, various substrates were investigated and the results are summarized in Scheme 2. The synthesis of products **2a**–**c** proceeded smoothly to give the corresponding β-hydroxylactones **2** in moderate to good yields with high diastereoselectivities, although **2d** was obtained in low yield. It may suggest that the existence of substituent(s) in γ- and/or δ-position of **2** help the formation of the intermediate structure which works in favor of the intramolecular cyclization. β-Substituted substrates on α,β-unsaturated ester moiety of **1** also gave the products (**2g** and **2h**) in low yields, but the formation of the 7-membered ring (**2f**) was not achieved. On the other hand, when the previous conditions using the RhCl(PPh_3_)_3_ catalyst was applied to the aldol-type cyclization, 5- and 6-membered products were obtained in good yields (see the yields and dr in parentheses in Scheme 2). However, the yields were also greatly affected by the substituents on the β-position of the α,β-unsaturated ester moiety, and all diastereomeric ratios were inferior in the case of the RhCl(PPh_3_)_3_ catalyst. The relative configurations of **2b** were confirmed by X-ray crystallography, and the relative configurations of **2c**, **2g**, and **2h** were confirmed by NOESY experiments.

### Mechanistic investigation of the intramolecular cyclization

**Scheme 3:**
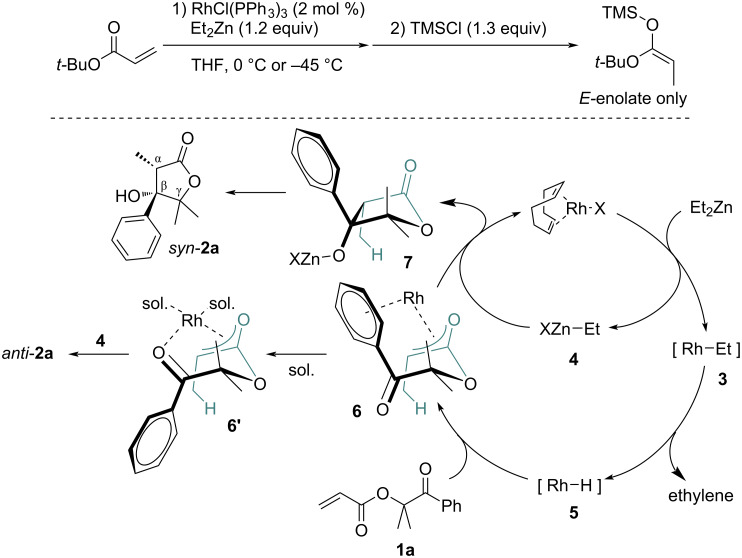
Detection of metal-enolate and proposed mechanism of intramolecular cyclization.

The reaction mechanism of the intramolecular cyclization can only be speculative at this stage. We have already reported the generation of a rhodium hydride (Rh–H) complex from RhCl(PPh_3_)_3_ and Et_2_Zn, in which the reaction with *tert*-butyl acrylate formed the corresponding *E*-silylenolate via 1,4-reduction at 0 °C [46], even if the reaction was performed at −45 °C (Scheme 3). Also in relation to this result, Mikami and his group reported a rhodium-catalyzed carboxylation of alkenes or activated alkenes by using CO_2_ with Et_2_Zn, and a similar Rh–H complex derived from [RhCl(cod)]_2_ and Et_2_Zn played an important role in this reaction [48]. Furthermore, Hopmann et al. detected the Rh–H complex derived from [RhCl(cod)]_2_ and Et_2_Zn by ^1^H NMR, and the detailed mechanism disclosed that the Rh–H complex did not interact with CO_2_ but with the benzene ring in the substrates through an η^6^ binding intermediate by DFT calculation [49].

Although another mechanism could not be denied in which a *Z*-enolate intermediate changes to an *E*-enolate under thermodynamic control, we propose the following mechanism on the basis of the above results (Scheme 3). The Rh–H complex **5** from [RhCl(cod)]_2_ and Et_2_Zn would generate predominantly the corresponding *E*-enolate **6** via 1,4-reduction, which is stabilized through η^6^ binding with benzene ring of the substrate. Subsequent transmetalation with zinc species **4** readily reacts with the carbonyl group to form the intramolecular C−C bonds at the α-position, then providing the product *syn*-**2a** with high regioselectivity. On the other hand, the use of higher coordinating solvents such as DMF or DMPU might break the weak η^6^ binding of rhodium complex to give *anti*-**2a**, predominantly.

### Synthesis of a chiral necic acid lactone of monocrotaline

**Figure 1:**
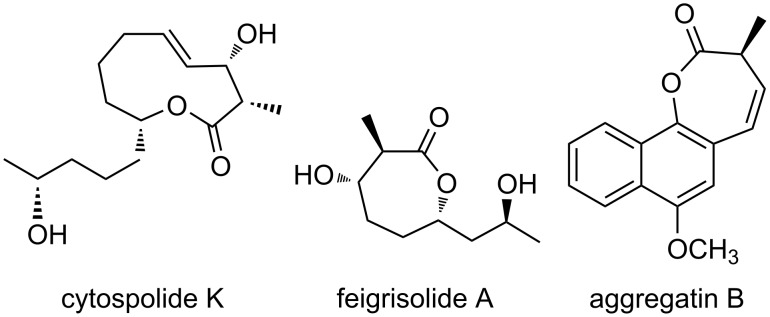
Bioactive natural products bearing a 3-hydroxy-2-methyllactone scaffold.

**Figure 2:**
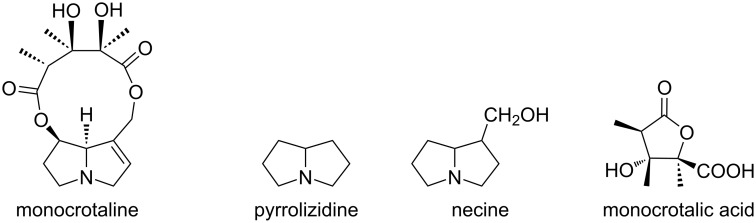
Monocrotaline and its structural components.

There are several reports of bioactive natural products that have a 3-hydroxy-2-methyllactone scaffold in the molecular structure. For example, cytospolide K2 [50] containing a 10-membered lactone and feigrisolide [51] containing a 7-membered lactone are known to exhibit cytotoxicity and antimicrobial activity. Moreover, antiviral activity was also confirmed for aggregatin B [52] containing a 7-membered lactone ring, in which the β-position hydroxy group was dehydrated (Figure 1). Monocrotaline is a kind of pyrrolizidine alkaloid and was isolated from seeds of *Crotalaria spectabilis* in 1935 [53]. Monocrotaline is used as compound for pulmonary hypertension model in rats. To date, some groups have reported synthetic methods and its synthetic supply will potentially contribute to hypertension treatment [54–57]. Although there have been a lot of reports of pyrrolizidine scaffolds or necine base, the synthesis of necic acid lactones such as monocrotalic acid is rare (Figure 2). Consequently, we attempted to apply the rhodium-catalyzed intramolecular reductive aldol-type reaction to the synthesis of a chiral necic acid lactone that is a part of structural component of monocrotaline.

**Scheme 4:**
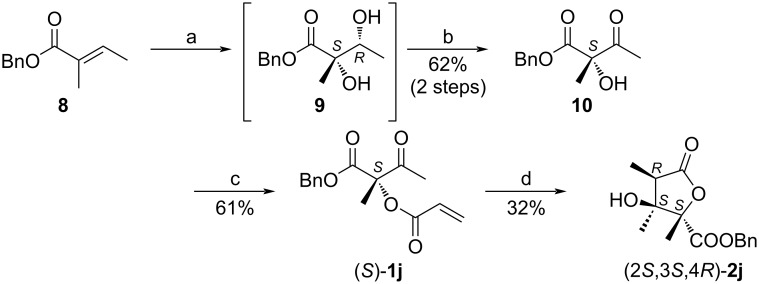
Synthetic route towards chiral necic acid lactone (2*S*,3*S*,4*R*)-**2j**. Conditions: a) CH_3_SO_2_NH_2_, AD-mix-β, *t*-BuOH, H_2_O. b) SO_3_·Py, Et_3_N, DMSO, CH_2_Cl_2_. c) DMAP, CH_2_Cl_2_, Et_3_N, acryloyl chloride, hydroquinone. d) [RhCl(cod)]_2_, THF, Et_2_Zn.

**Figure 3:**
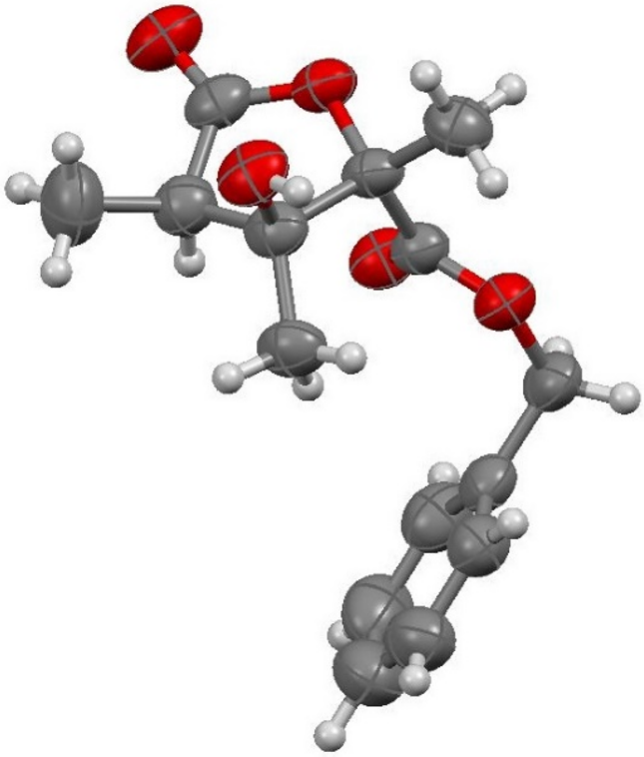
Molecular structure of necic acid lactone (2*S*,3*S*,4*R*)-**2j** in the crystal.

According to the literature, a Sharpless dihydroxylation of benzyl tiglate (**8**) to form a chiral diol **9** was followed by a Parikh–Doering oxidation to give the corresponding product **10** in 62% yield (Scheme 4) [58–59]. Subsequent acryloylation in the presence of DMAP and hydroquinone gave the intramolecular cyclization starting material (*S*)-**1j** in 61% yield. The transformation of the compound (*S*)-**1j** in the rhodium-catalyzed intramolecular reductive aldol-type cyclization proceeded smoothly and gave the chiral necic acid lactone (2*S*,3*S*,4*R*)-**2j** in 32% yield (Figure 3).

## Conclusion

In conclusion, during the development of a rhodium-catalyzed intramolecular reductive cyclization, we found that using [RhCl(cod)]_2_ improved the diastereomeric ratio of the products compared with other Rh catalysts. It seems that using [RhCl(cod)]_2_ leads to milder reaction conditions that lead to highly improved diastereomeric ratios. In addition, we demonstrated a new approach to a necic acid lactone **2j** that is a diastereomer of monocrotalic acid, a key intermediate of monocrotalin.

## Supporting Information

File 1 General procedures and analytical data, including copies of ^1^H NMR, ^13^C NMR, and X-ray crystallography.
