# Supplementary material for: Rhodium-catalyzed intramolecular reductive aldol-type cyclization: Application for the synthesis of a chiral necic acid lactone
Source: Beilstein J Org Chem. 2022 Dec 2;18:1642–8. doi: 10.3762/bjoc.18.176 (PMC9727276; doi:10.3762/bjoc.18.176)

BEILSTEIN JOURNAL OF ORGANIC CHEMISTRY

Supporting Information

for

Rhodium-catalyzed intramolecular reductive aldol-type cyclization: Application for the synthesis of a chiral necic acid lactone

Motoyuki Isoda, Kazuyuki Sato, Kenta Kameda, Kana Wakabayashi, Ryota Sato, Hideki Minami, Yukiko Karuo, Atsushi Tarui, Kentaro Kawai and Masaaki Omote

Beilstein J. Org. Chem. **2022,** *18,* 1642–1648. doi:10.3762/bjoc.18.176

General procedures and analytical data, including copies of ^{1}H NMR, ^{13}C NMR, and X-ray crystallography

License and Terms: This is a supporting information file under the terms of the Creative Commons Attribution License (https://creativecommons.org/licenses/by/4.0). Please note that the reuse, redistribution and reproduction in particular requires that the author(s) and source are credited and that individual graphics may be subject to special legal provisions.
The license is subject to the *Beilstein Journal of Organic Chemistry* terms and conditions: (https://www.beilstein-journals.org/bjoc/terms)

Table of contents

General information …………………………………………………. S2

Materials ……………………………………………….…………… S2

Experimental section

General procedure for the cyclization precursor (**1**). ..……….. S2

General procedure for intramolecular

reductive aldol-type cyclization. ………..…S3

Spectroscopic data ………………………………….…………... S4–S6

Thermal ellipsoid plot of the crystallographic structure. …….. S7–S14

References …….…………………………..…………………….. S15

NMR charts ………………………………………….…..… S16–S24

General information:

^{1}H NMR and ^{13}C NMR spectra were recorded on JEOL JNM-ECZS-400 spectrometers. Chemical shifts of ^{1}H NMR are reported in ppm from signal of tetramethylsilane (TMS: 0 ppm) as an internal standard. Chemical shifts of ^{13}C NMR are reported in ppm from signal of $CDCl_3$ (77.20 ppm) as an internal standard. All data are reported as follows: chemical shifts, relative integration value, multiplicity (s = singlet, d = doublet, t = triplet, q = quartet, br s= broad singlet, m = multiplet), coupling constants (Hz). Mass spectra were obtained on JEOL JMS-700T spectrometers. Melting points were measured on Yanagimoto micro melting point apparatus MP-S3.

Materials:

Dichloromethane (DCM) was distilled over phosphorus pentoxide just before use. Tetrahydrofuran (THF) was purchased from Kanto Chemical Co. Inc. as "Dehydrated". All other solvents were distilled under inert argon atmosphere or under reduced pressure before use. All commercially available reagents were used without further purification. All experiments were carried out under argon atmosphere in flame-dried glassware using standard inert techniques for introducing reagents unless otherwise noted.

Experimental section:

General procedure for the cyclization precursor (1).

To a solution of hydroxyketone (5 mmol) in DCM (50 mL) was added Et_3N (8 mmol) and stirred for 30 min. Then, α,β-unsaturated acid chloride (6 mmol) was added to the mixture and stirred until TLC analysis showed the reaction to be complete. The mixture was quenched with 10% HCl and extracted with DCM. The organic layer was washed with sat. NaCl and dried over $MgSO_4$. The solvent was removed in vacuo to give the crude mixture. The mixture was purified by recrystallization from hexane and to obtain the product **1**.

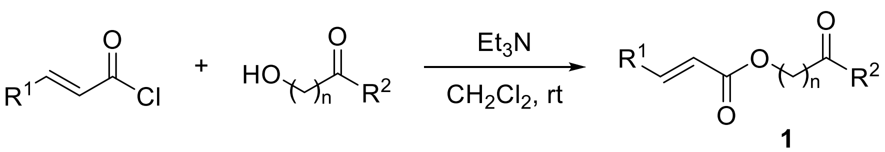

General procedure for intramolecular reductive aldol-type cyclization.

To a solution of $[RhCl(cod)]_2$ (2 mol %) in DCM (2.5 mL) was added cyclization precursor (**1**; 1 mmol) at 0 °C, and then 1.0 M Et_2Zn in hexane (1.2 mL) was gradually added to the mixture. After consumption of the starting material **1** as monitored by TLC, the mixture was quenched with 10% HCl and extracted with AcOEt. The organic layer was washed with sat. NaCl and dried over $MgSO_4$. The solvent was removed in vacuo, and the residue was purified by column chromatography (SiO_2; AcOEt/hexane 4:1) to give the product.

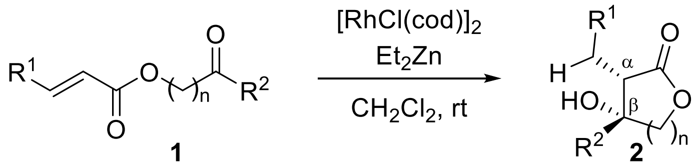

Synthesis for a chiral necic acid lactone.

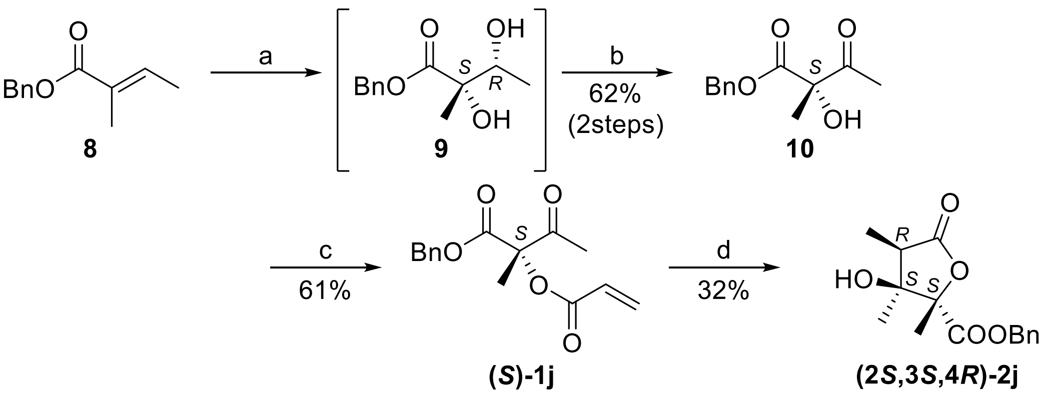

a) $CH_3SO_2NH_2$, AD-mix-β, *t*-BuOH, H_2O. b) SO_3•Py, Et_3N, DMSO, CH_2Cl_2.
c) DMAP, CH_2Cl_2, Et_3N, acryloyl chloride, hydroquinone. d) $[RhCl(cod)]_2$, THF, Et_2Zn.

Benzyl (*S*)-2-hydroxy-2-methyl-3-oxobutanoate (10):

Under an Ar atomosphere, to a solution of $CH_3SO_2NH_2$ (4.5 mmol) and AD-mix-β (15 g) in *t*-BuOH/H_2O 1:1 (18 mL) was stirred for 15 min, then the mixture was cooled to 0 °C. Benzyl tiglate (**8**, 9 mmol) was added to the mixture and it was stirred for 16 h at the same temperature. After consumption of starting material **8** as monitored by TLC, the mixture was quenched with sat. NH_4Cl and extracted with DCM. The organic layer was washed with sat. NaCl and dried over $MgSO_4$. The solvent was removed in vacuo, and the residue was passed through a short column (SiO_2; AcOEt/hexane 3:7) to correct **9**.

Under an Ar atomosphere, to a solution of corrected **9** in DCM (30 mL) was added DMSO (5.5 mL) and Et_3N (45 mmol) and then a solution of SO_3·Py (28 mmol) in DMSO (14 mL) was added to the mixture. The resulting mixture was stirred for 24 h at ambient temperature, then it was quenched with sat. NH_4Cl and extracted with DCM. The organic layer was washed with sat. NaCl and dried over $MgSO_4$. The solvent was removed in vacuo, and the residue was purified by column chromatography (SiO_2; AcOEt/hexane 1:4) to give the product **10** in 62% (1.25g, 5.6 mmol, 96% ee) from **8**.

Benzyl (*S*)-2-(acryloyloxy)-2-methyl-3-oxobutanoate ((*S*)-1j):

Under an Ar atomosphere, to a solution of DMAP (0.23 mmol) in DCM (2 mL) was added **5** (2.23 mmol) and Et_3N (6.5 mmol) at 0 °C and the mixture was stirred for 30 min at the same temperature. To the mixture was added acryloyl chloride (4.9 mmol) and hydroquinone (0.5 mmol) and stirred overnight at the same temperature. The mixture was quenched with sat. NH_4Cl and extracted with DCM. The organic layer was washed with sat. NaCl and dried over $MgSO_4$. The solvent was removed in vacuo, and the residue was passed through a short column (SiO_2; AcOEt/hexane 1:4) to give the product (*S*)-**1j** in 61% (378 mg, 1.37 mmol) from **3**.

Benzyl (2*S*,3*S*,4*R*)-3-hydroxy-2,3,4-trimethyl-5-oxotetrahydrofuran-2-carboxylate (2j):

To a solution of $[RhCl(cod)]_2$ (2 mol %) in THF (3.25 mL) was added cyclization precursor ((*S*)-**1j**; 1.3 mmol) at ambient temperature, and then 1.0 M Et_2Zn in hexane (1.56 mL) was gradually added to the mixture and the mixture was stirred for 48 h. The mixture was quenched with 10% HCl and extracted with AcOEt. The organic layer was washed with sat. NaCl and dried over $MgSO_4$. The solvent was removed in vacuo, and the residue including the diastereomeric mixture was purified by column chromatography (AcOEt/toluene 4:1) to give the product (2*S*,3*S*,4*R*)-**2j** in 32% (116 mg, 0.41 mmol).

Spectroscopic data:

Benzyl (*S*)-2-(acryloyloxy)-2-methyl-3-oxobutanoate ((*S*)-1j)

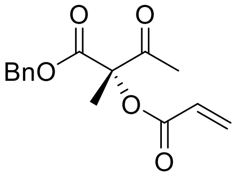

Pale yellow oil; ^{1}H NMR (400 MHz, $CDCl_3$) δ: 1.77 (s, 3H), 2.33 (s, 3H), 5.20 (s, 2H), 5.95 (dd, J = 10.5, 1.2 Hz, 1H), 6.21 (dd, J = 17.3, 10.5 Hz, 1H), 6.49 (dd, J = 17.3, 1.1 Hz, 1H), 7.30–7.38 (5H, m); MS *m/z*: 276 (M^+). HR-MS Calcd for $C_{15}H_{16}O_5$: 276.0998 (M^+), Found: 276.0992; IR (neat) cm^{-1}: 1193, 1267, 1634, 1724, 3462.

(3*R*,4*R*/3*S*,4*S*)-4-Hydroxy-3,5,5-trimethyl-4-phenyldihydrofuran-2(3*H*)-one (*syn*-2a)[1]

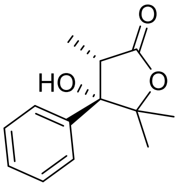

A colorless solid; mp 142–146°C; ^{1}H NMR (400 MHz, $CDCl_3$) δ: 1.03 (s, 3H), 1.23 (d, J = 7.2 Hz, 3H), 1.56 (s, 3H), 2.04 (s, 1H), 3.60 (q, J = 7.2 Hz, 1H), 7.35–7.49 (m, 5H); ^{13}C NMR (100 MHz, $CDCl_3$) δ: 7.59, 20.17, 25.40, 42.30, 83.33, 88.44, 126.4, 128.9, 129.0, 138.4, 176.7; HR-MS Calcd for $C_{13}H_{16}O_3$: 220.1099 (M^+), Found: 220.1096; IR (KBr) cm^{-1}: 3439, 1749.

(3*R*,4*S*/3*S*,4*R*)-4-Hydroxy-3,5,5-trimethyl-4-phenyldihydrofuran-2(3*H*)-one (*anti*-2a)[1]

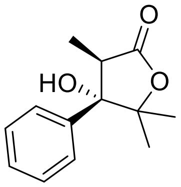

A colorless solid; mp 185–187°C; ^{1}H NMR (400 MHz, $CDCl_3$) δ: 0.99 (d, *J* = 7.2 Hz, 3H), 1.01 (s, 3H), 1.60 (s, 3H), 2.41 (s, 1H), 3.28 (q, *J* = 7.2 Hz, 1H), 7.28–7.40 (m, 5H); ^{13}C NMR (100 MHz, $CDCl_3$) δ: 8.92, 23.82, 24.22, 46.03, 83.58, 87.93, 126.1, 128.1, 128.7, 138.9, 176.7; HR-MS Calcd for $C_{13}H_{16}O_3$: 220.1099 (M^+), Found: 220.1105; IR (KBr) cm^{-1}: 3439, 1749.

(3*S*,4*S*,5*R*/3*R*,4*R*,5*S*)-4-Hydroxy-3-methyl-4,5-diphenyldihydrofuran-2(3*H*)-one (Major form) (2b)

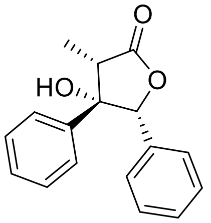

A colorless solid; mp: 150.5–152.5 °C; ^{1}H NMR (400 MHz, $CDCl_3$) δ: 1.19 (d, *J* = 6.86 Hz, 3H), 1.68 (d, *J* = 1.37 Hz, 1H), 3.19 (qd, *J* = 5.49, 1.37 Hz, 1H), 5.80 (s, 1H), 7.06–7.09 (m, 2H), 7.27–7.46 (m, 8H); ^{13}C NMR ($CDCl_3$) δ: 7.0, 49.0, 81.6, 86.9, 125.6, 126.7, 128.4, 128.8, 129.0, 129.3, 132.0, 139.1, 176.7; HR-MS Calcd for $C_{17}H_{16}O_3$: 268.1099 (M^+), Found: 268.1098; IR (KBr) cm^{-1}: 698, 942, 1449, 1772, 3468.

4-Hydroxy-3-methyl-4-phenyldihydrofuran-2(3*H*)-one (*syn*-form) (2c)

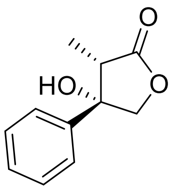

A colorless oil; ^{1}H NMR ($CDCl_3$) δ: 1.18 (d, *J* = 7.32 Hz, 3H), 3.00 (q, *J* = 7.32 Hz, 1H), 3.15 (br s, 1H), 4.35 (d, *J* = 10.1 Hz, 1H), 4.39 (d, *J* = 10.1 Hz, 1H), 7.33–7.50 (m, 5H); ^{13}C NMR ($CDCl_3$) δ: 6.9, 45.7, 78.3, 79.9, 125.3, 128.5, 129.0, 139.5, 178.2; HR-MS Calcd for $C_{11}H_{12}O_3^+$: 192.0786 (M^+), Found: 192.0790 (M^+); IR (neat) cm^{-1}: 701, 1187, 1772, 2983, 3448.

4-Hydroxy-3,4-dimethyltetrahydro-2*H*-pyran-2-one (Diastereomeric mixture) (2d)

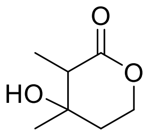

A colorless oil; ^{1}H NMR ($CDCl_3$) δ: 1.26 (d, *J* = 7.32 Hz, 0.16H),1.27 (s, 0.16H), 1.31 (d, *J* = 7.32 Hz, 2.84H), 1.38 (s, 2.84H), 1.93–2.14 (m, 3H), 2.46 (q, *J* = 7.32 Hz, 0.95H), 2.67 (q, *J* = 7.32 Hz, 0.05H), 4.25–4.31 (m, 1H), 4.47–4.57 (m, 1H); ^{13}C NMR ($CDCl_3$) δ: 9.2, 11.7, 25.4, 28.3, 36.3, 37.4, 46.2, 47.7, 65.0, 65.3, 70.2, 70.8, 174.0, 174.7; HR-MS (FAB$^+$) Calcd for $C_7H_{13}O_3^+$: 145.0865 $[M+H]^+$, Found: 145.0874 $[M+H]^+$; IR (neat) cm^{-1}: 740, 930, 1242, 1433, 1587, 2916, 3017.

4-Hydroxy-3,4-dimethylchroman-2-one (Diastereomeric mixture) (2e)

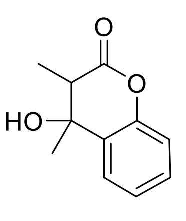

A yellow oil; ^{1}H NMR (CDCl$_3$) δ: 1.32 (d, *J* = 7.32 Hz, 1.2H), 1.34 (d, *J* = 6.86 Hz, 1.8H), 1.43 (s, 1.8H), 1.64 (s, 1.2H), 2.65 (q, *J* = 7.32 Hz, 0.4H), 2.93 (q, *J* = 6.86Hz, 0.6H,), 7.05–7.09 (m, 1H), 7.18–7.22 (m, 1H), 7.30–7.37 (m, 1H), 7.50–7.56 (m, 1H); ^{13}C NMR (CDCl$_3$) δ: 9.30, 10.0, 23.8, 26.6, 46.4, 46.9, 70.9, 71.8, 117.0, 124.4, 124.7, 125.1, 128.7, 129.8, 130.1, 131.2, 149.2, 149.9, 170.6; HR-MS Calcd for $C_{11}H_{12}O_3^+$: 192.0786 (M$^+$), Found: 192.0791 (M$^+$); IR (neat) cm^{-1}: 761, 1207, 1454, 1766, 3420.

3-Ethyl-4-hydroxy-5,5-dimethyl-4-phenyldihydrofuran-2(3*H*)-one (*syn*-form) (2g)

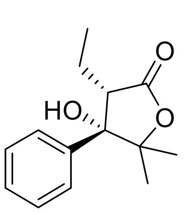

A colorless solid; Mp: 108.9–109.1 °C; ^{1}H NMR (CDCl$_3$) δ: 1.04 (s, 3H), 1.11 (t, *J* = 7.55 Hz, 3H), 1.45–1.55 (m, 1H), 1.50 (s, 3H), 1.84–1.95 (m, 1H), 2.07 (s, 1H), 3.35 (dd, *J* = 5.49, 8.23, 1H), 7.35–7.44 (m, 3H), 7.47–7.50 (m, 2H); ^{13}C NMR (CDCl$_3$) δ: 13.0, 17.8, 20.0, 25.6, 48.7, 83.7, 88.0, 126.4, 128.7, 128.8, 138.7, 176.5; HR-MS (FAB$^+$) for $C_{14}H_{19}O_3^+$: 235.1334 [M+H]$^+$, Found: 235.1339 [M+H]$^+$; IR (KBr) cm^{-1}: 703, 756, 1758, 3003, 3459.

3-Benzyl-4-hydroxy-5,5-dimethyl-4-phenyldihydrofuran-2(3*H*)-one (*syn*-form) (2h)

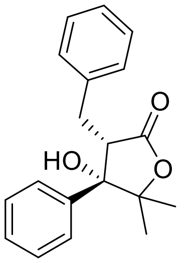

A colorless solid; Mp: 184.0–184.3 °C; ^{1}H NMR (CDCl$_3$) δ: 1.02 (s, 3H), 1.51 (s, 3H), 2.23 (s, 1H), 2.78 (dd, *J* = 14.4, 4.8 Hz, 1H), 3.21 (q, *J* = 7.0 Hz, 1H), 3.72 (dd, *J* = 7.1, 4.8 Hz, 1H), 7.27–7.13 (m, 5H), 7.36–7.30 (m, 5H); ^{13}C NMR (CDCl$_3$) δ: 20.1, 25.5, 30.5, 49.7, 84.2, 88.3, 126.4, 126.7, 128.6, 128.7 (2C), 129.4, 138.0, 139.2, 176.0; HR-MS for $C_{19}H_{20}O_3^+$: 296.1412 (M$^+$), Found: 296.1407 (M$^+$); IR (KBr) cm^{-1}: 697, 1056, 1560, 1752, 3482.

Benzyl (2*S*,3*S*,4*R*)-3-hydroxy-2,3,4-trimethyl-5-oxotetrahydrofuran-2-carboxylate ((2*S*,3*S*,4*R*)-2j)

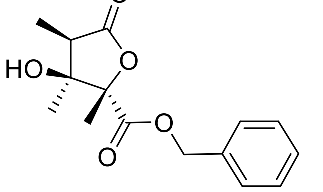

A colorless solid; ^{1}H NMR (400 MHz, CDCl$_3$) δ: 1.16 (s, 3H), 1.17 (d, *J* = 7.2 Hz, 3H), 1.63 (s, 3H), 1.74 (s, 1H), 2.72 (q, *J* = 7.2 Hz, 1H), 5.16 (d, *J* = 11.9 Hz, 1H), 5.25 (d, *J* = 11.9 Hz, 1H), 7.33–7.40 (5H, m); ^{13}C NMR (100 MHz, CDCl$_3$) δ: 7.37, 16.5, 21.5, 44.2, 68.1, 79.0, 89.2, 128.8, 128.9, 129.0, 134.7, 170.9, 177.4; MS *m/z*: 278 (M$^+$). HR-MS Calcd for $C_{15}H_{18}O_5$: 278.1154 (M$^+$), Found: 278.1156; IR (KBr) cm^{-1}: 1458, 1734, 3431.

Thermal ellipsoid plot of the crystallographic structure:

X-ray crystallographic analysis of *syn*-2a:

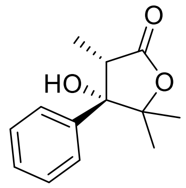

The product was recrystallized from ethyl acetate/hexane. The single crystal was mounted on a glass fiber. All measurements were made on a Rigaku R-AXIS RAPID diffractometer using graphite monochromated CuKα (λ = 1.54187 Å) radiation. The 2θ (max) value cut of 68.01°. The crystal structure was solved by SHELXT[2] and refined by SHELXL[3]. The crystallographic data were summarized in the following table.

emprical formula	$C_{26}H_{32}O_6$
formula weight	440.54
crystal system	Triclinic
space group	$P\bar{1}$(2)
a, Å	8.5541(5)
b, Å	11.5651(7)
c, Å	12.9776(7)
α, °	97.656(7)
β, °	100.498(7)
γ, °	104.252(7)
V, Å^3	1201.96(13)
Z	2
D_{calc}, g/cm^3	1.217
T, °C	23
μ (CuKα), cm^{-1}	0.698
No. of reflens measured	13308
No. of reflens observed	2169
No. of reflens variable	319
R (All reflections)	0.0583
R_w (All reflections)	0.0797
Good of Fit	1.085
Max Shift/Error	0.000

The crystal structure has been deposited at the Cambridge Crystallographic Data Centre (CCDC 2109686). The data can be obtained free of charge via the Internet at www.ccdc.cam.ac.uk/conts/retrieving.html.

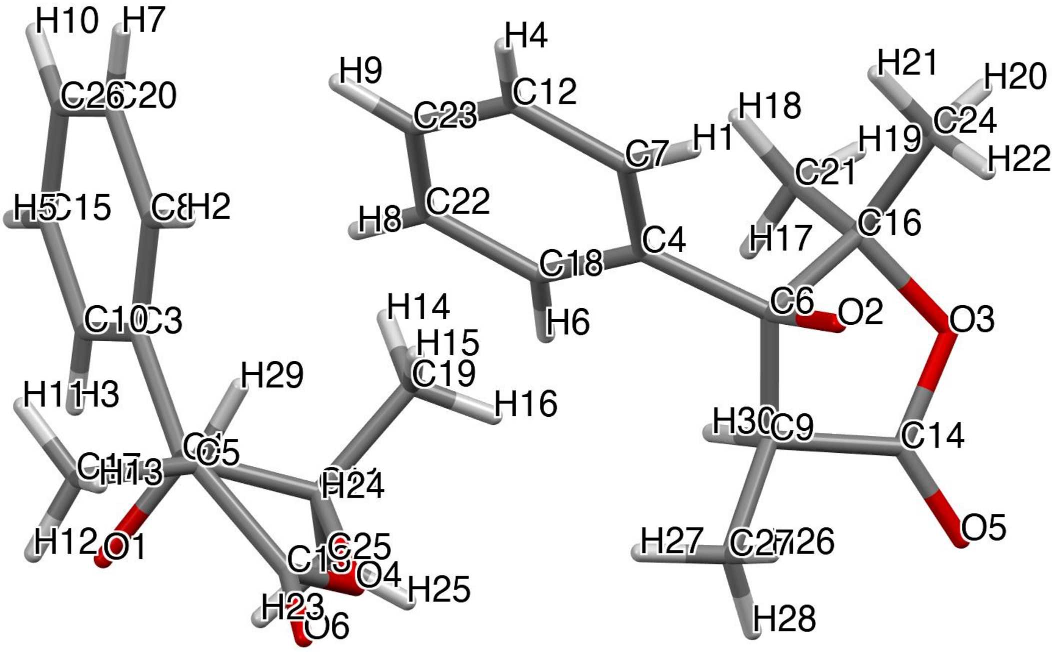
H10
H7
H4
H9
H21
H20
C12
H18
C23
C24
H1
H19
C7
H22
C21
C22
H8
C16
C4
H17
C18
H6
C6
O2
O3
H14
H15
H29
C19
H16
C14
O5
H24
H27
H28
H25
O6
O1
H12
H23

X-ray crystallographic analysis of *anti*-2a:

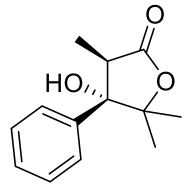

The product was recrystallized from ethyl acetate/hexane. The single crystal was mounted on a glass fiber. All measurements were made on a Rigaku R-AXIS RAPID diffractometer using graphite monochromated CuKα (λ = 1.54187 Å) radiation. The 2θ (max) value cut of 68.14°. The crystal structure was solved by SHELXT[2]and refined bySHELXL[3]. The crystallographic data were summarized in the following table.

emprical formula	$C_{13}H_{16}O_3$
formula weight	220.27
crystal system	orthorhombic
space group	Iba2(45)
a, Å	13.6211(6)
b, Å	25.5188(12)
c, Å	6.8860(3)
β, °	90.0000
V, Å^3	2393.53(19)
Z	8
D_{calc}, g/cm^3	1.222
T, °C	23
μ (CuKα), cm^{-1}	0.701
No. of reflens measured	12470
No. of reflens observed	1204
No. of reflens variable	168
R (All reflections)	0.0447
R_w (All reflections)	0.0372
Good of Fit	1.068
Max Shift/Error	0.072

The crystal structure has been deposited at the Cambridge Crystallographic Data Centre (CCDC 2109703). The data can be obtained free of charge via the Internet at www.ccdc.cam.ac.uk/conts/retrieving.html.

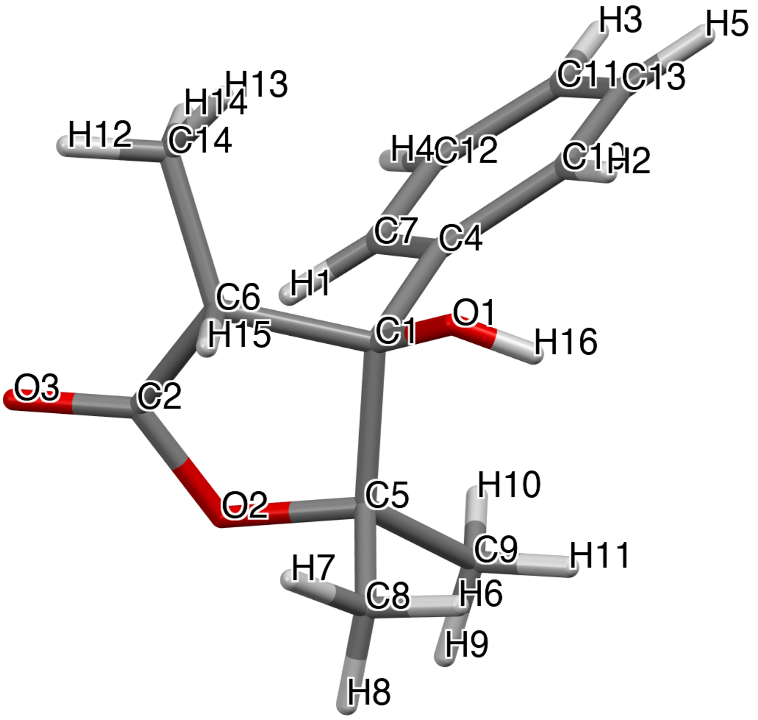
H3
H5
C11
C13
H13
H14
H12
C14
H4
C12
H2
C7
C4
H1
C6
O1
H15
C1
H16
O3
C2
O2
C5
H10
C9
H11
H7
C8
H6
H9
H8

X-ray crystallographic analysis of 2b (major form):

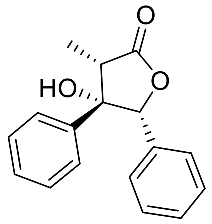

The product was recrystallized from ethyl acetate/hexane. The single crystal was mounted on a glass fiber. All measurements were made on a Rigaku R-AXIS RAPID diffractometer using graphite monochromated CuKα (λ = 1.54187 Å) radiation. The 2θ (max) value cut of 68.09°. The crystal structure was solved by SHELXT[2] and refined by SHELXL[3]. The crystallographic data were summarized in the following table.

emprical formula	$C_{17}H_{16}O_3$
formula weight	268.31
crystal system	monoclinic
space group	C 2/c(15)
a, Å	27.7420(10)
b, Å	6.3547(2)
c, Å	18.7633(7)
β, °	119.894(8)
V, Å^3	2867.7(3)
Z	8
D_{calc}, g/cm^3	1.243
T, °C	23
μ (CuKα), cm^{-1}	0.685
No. of reflens measured	15128
No. of reflens observed	2376
No. of reflens variable	200
R (All reflections)	0.0459
R_w (All reflections)	0.0708
Good of Fit	1.614
Max Shift/Error	0.020

The crystal structure has been deposited at the Cambridge Crystallographic Data Centre (CCDC 2109704). The data can be obtained free of charge via the Internet at www.ccdc.cam.ac.uk/conts/retrieving.html.

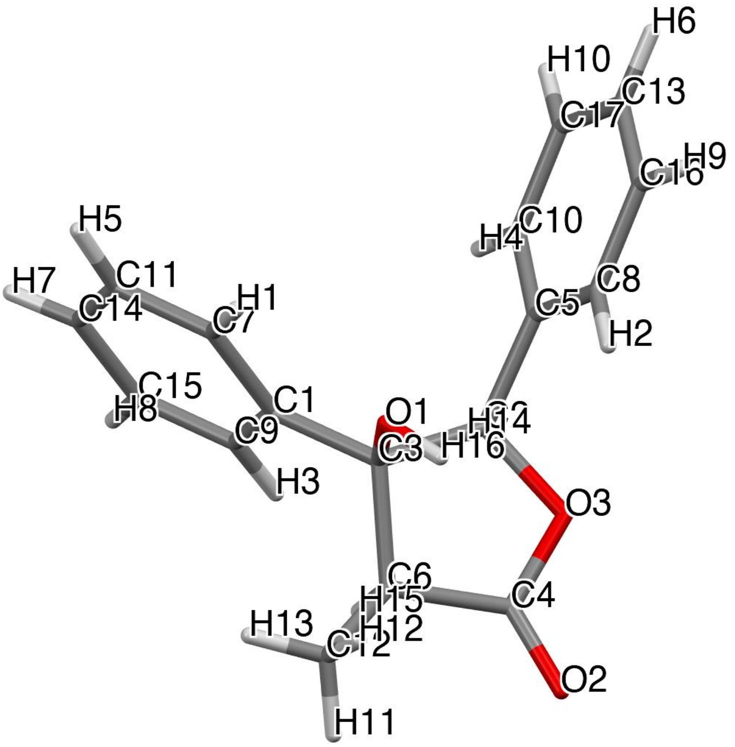
H6
H10
C13
C17
H9
C16
H5
C10
H4
H7
C11
C8
C14
H1
C5
C7
H2
C15
C1
O1
H8
C9
H14
C3
H16
H3
O3
C6
C4
H15
H13
H12
C12
O2
H11

X-ray crystallographic analysis of (2*S*,3*S*,4*R*)-2j:

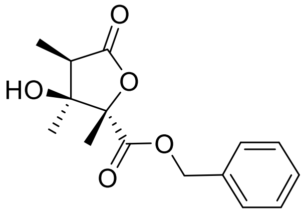

The product was recrystallized from ethyl acetate/hexane. The single crystal was mounted on a glass fiber. All measurements were made on a Rigaku R-AXIS RAPID diffractometer using graphite monochromated CuKα (λ = 1.54187 Å) radiation. The 2θ (max) value cut of 136.4°. The crystal structure was solved by SHELXT[2]and refined by SHELXL[3]. The crystallographic data were summarized in the following table.

emprical formula	$C_{15}H_{18}O_5$
formula weight	278.30
crystal system	monoclinic
space group	P 2_1(4)
a, Å	7.7895(5)
b, Å	10.8470(6)
c, Å	8.8801(5)
β, °	91.079(6)
V, Å^3	750.17(8)
Z	2
D_{calc}, g/cm^3	1.232
T, °C	23
μ (CuKα), cm^{-1}	0.770
No. of reflens measured	8338
No. of reflens observed	2521
No. of reflens variable	189
R (All reflections)	0.0369
R_w (All reflections)	0.1142
Good of Fit	0.839
Max Shift/Error	0.004

The crystal structure has been deposited at the Cambridge Crystallographic Data Centre (CCDC 2111153). The data can be obtained free of charge via the Internet at www.ccdc.cam.ac.uk/conts/retrieving.html.

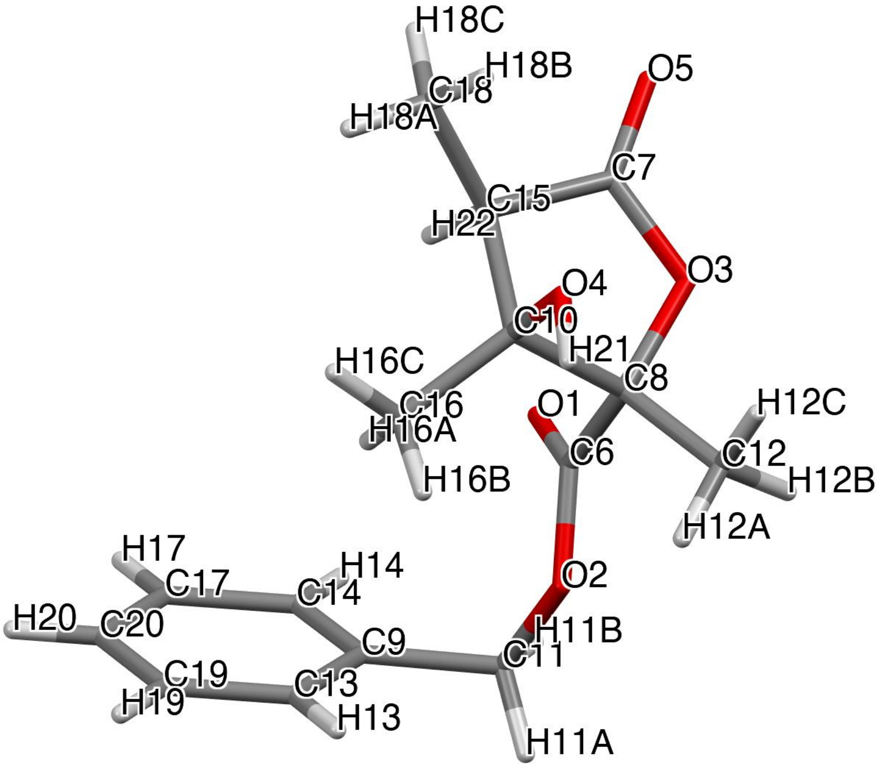
H18C
H18B
O5
C18
H18A
C7
C15
H22
O3
O4
C10
H21
H16C
C8
C16
O1
H12C
H16A
C6
C12
H16B
H12B
H12A
H17
H14
O2
C17
C14
H20
C20
H11B
C9
C11
C19
C13
H19
H13
H11A

References:

[1] Isoda, M.; Sato, K.; Tokura, Y.; Tarui, A.; Omote, M.; Ando, A. *Chem. Pharm. Bull.* 2014, 62, 956–961.

[2] Sheldrick, G. M. (2014) SHELXT Version 2014/5, *Acta Cryst*. A70, C1437.

[3] Sheldrick, G.M. (2008) SHELXL Version 2018/1, *Acta Cryst*. A64, 112-122.

NMR charts:

^{1}H NMR (400 MHz) and ^{13}C NMR (100 MHz) spectra of *syn*-2a (CDCl$_3$)

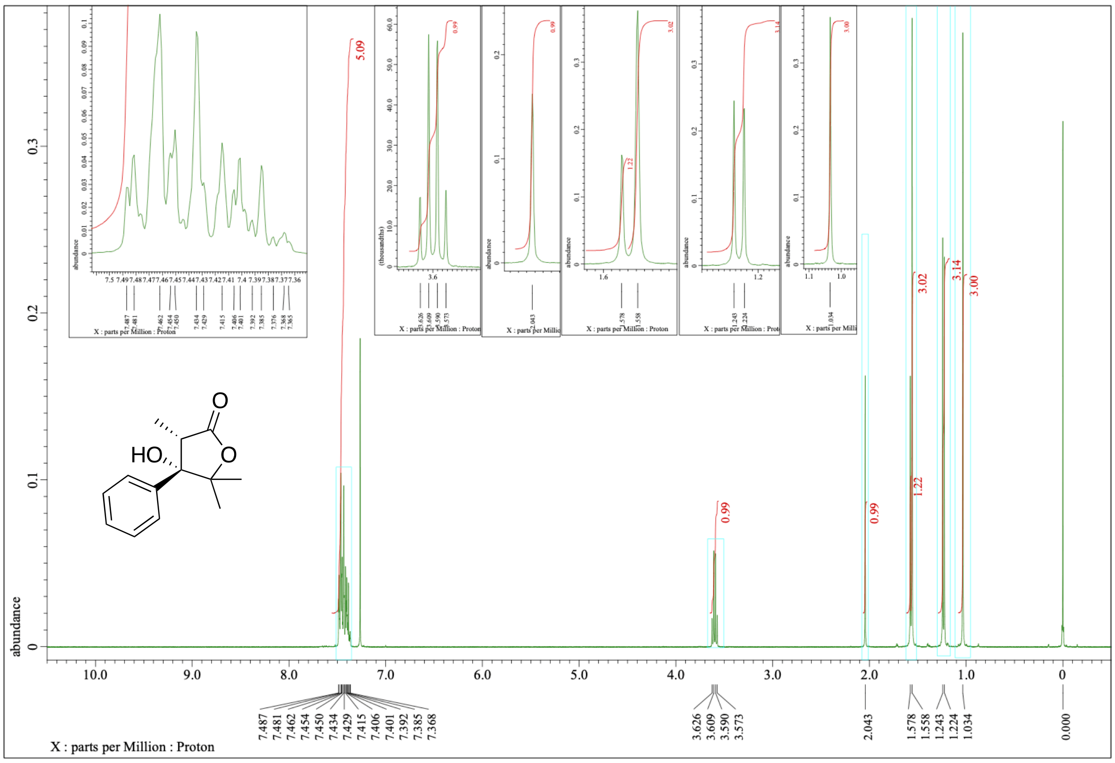

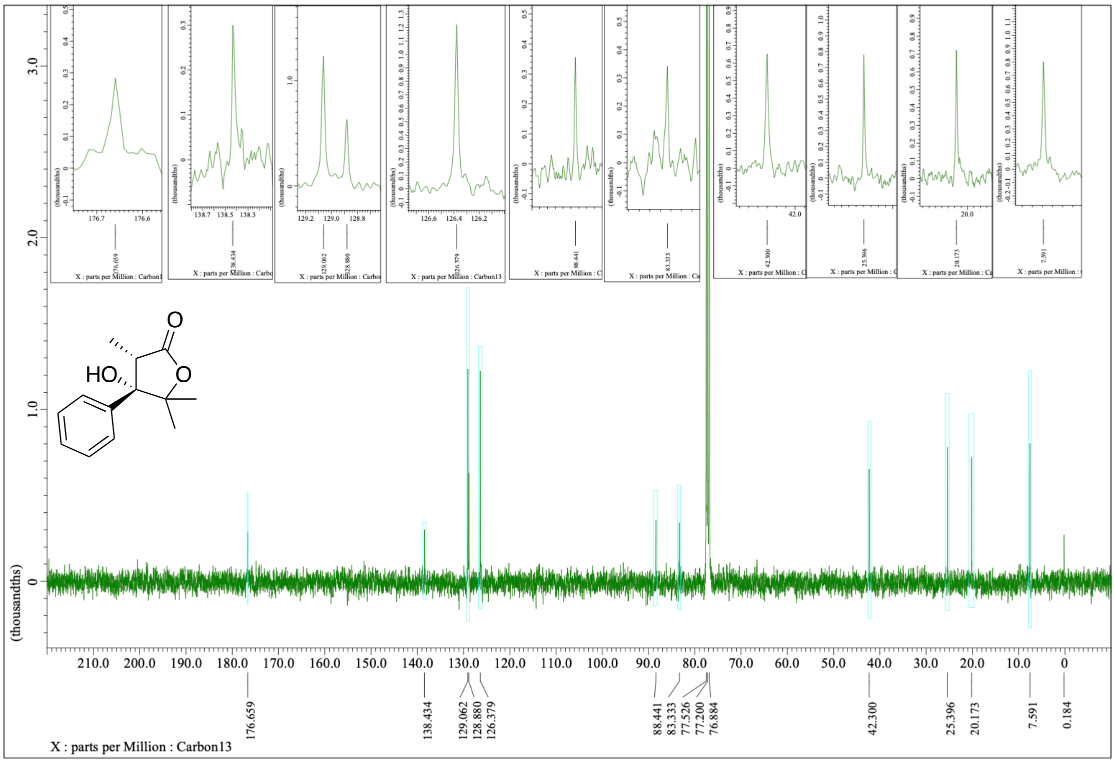

^{1}H NMR (400 MHz) and ^{13}C NMR (100 MHz) spectra of *anti*-2a ($CDCl_3$)

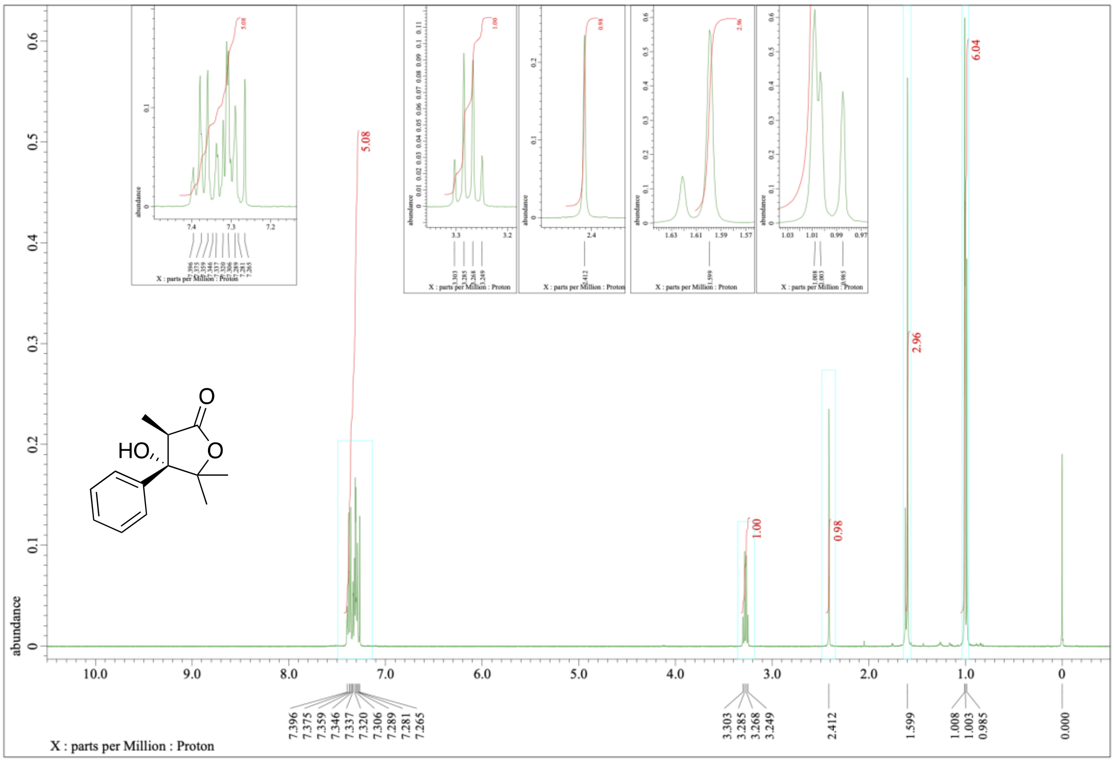

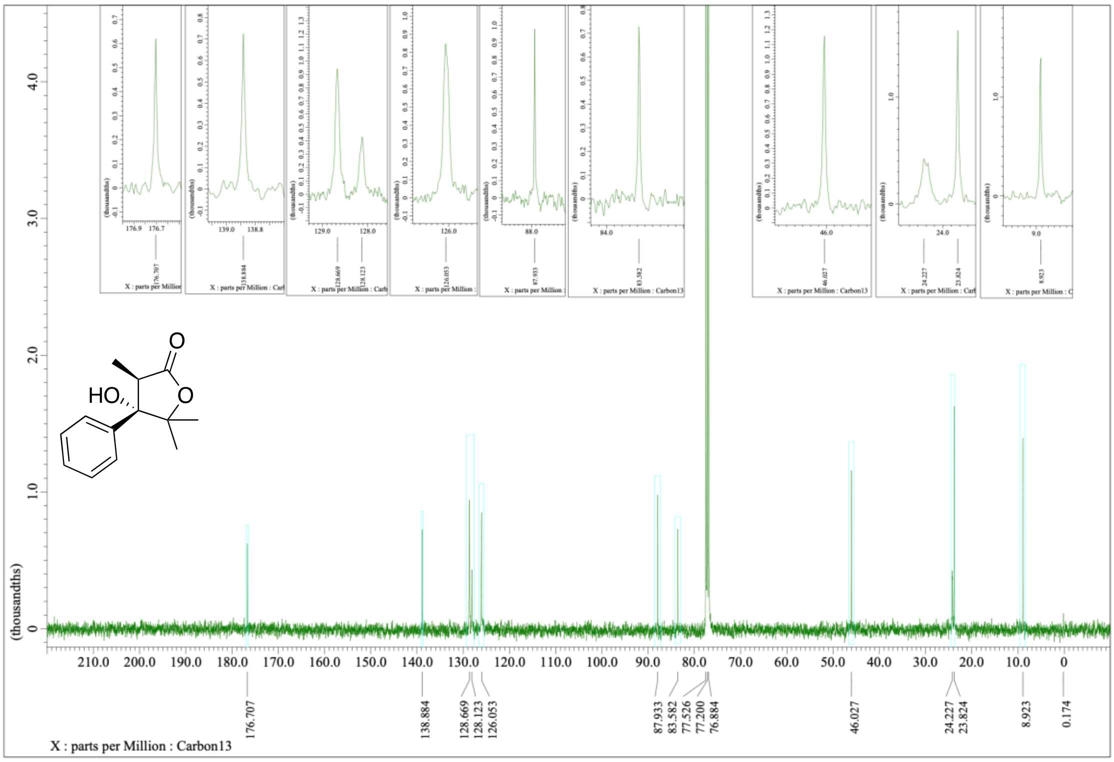

^{1}H NMR (400 MHz) and ^{13}C NMR (100 MHz) spectra of 2b ($CDCl_3$)

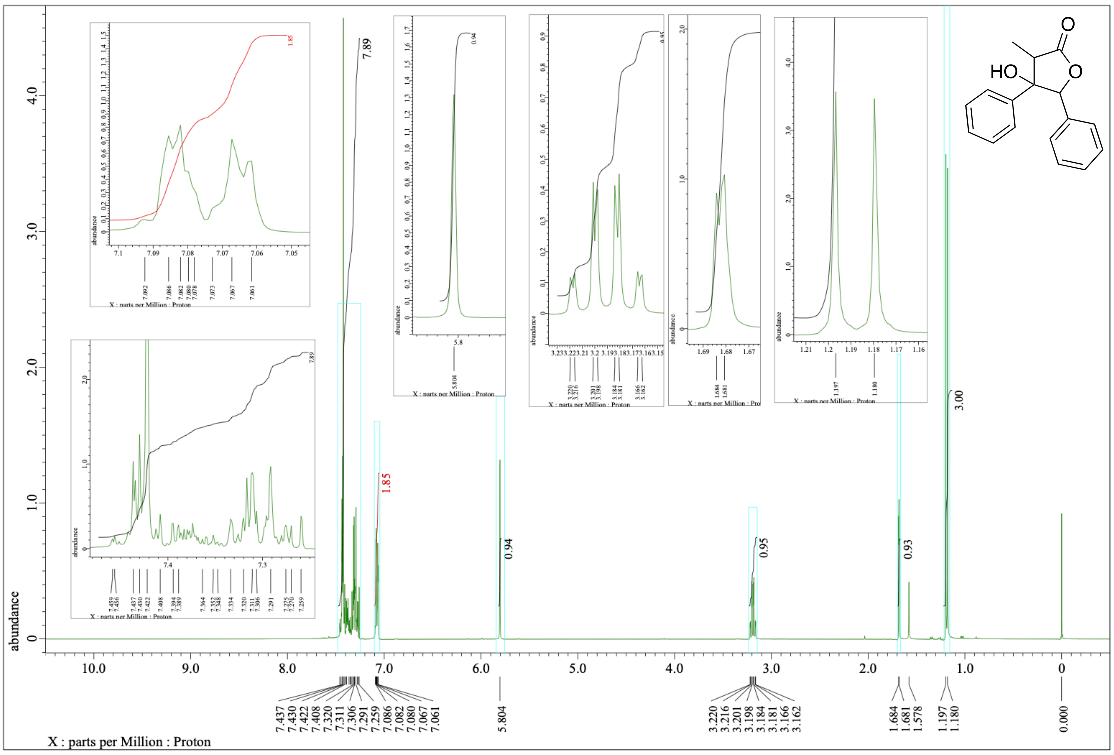

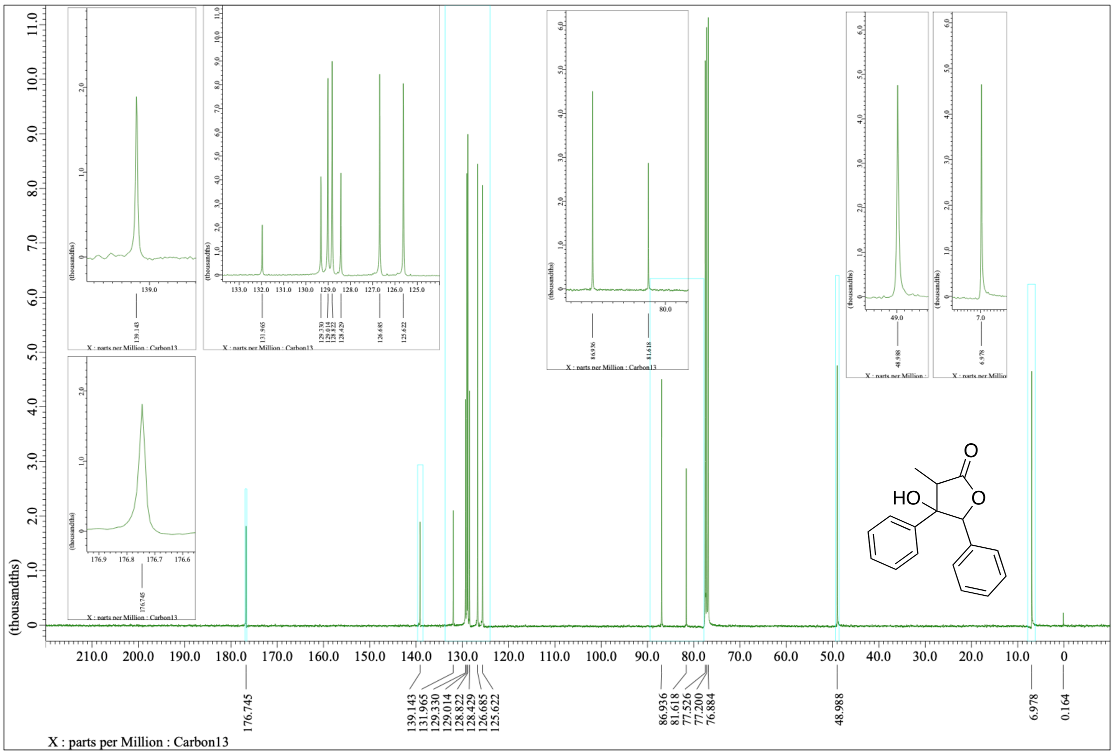

^{1}H NMR (400 MHz) and ^{13}C NMR (100 MHz) spectra of 2c (CDCl3)

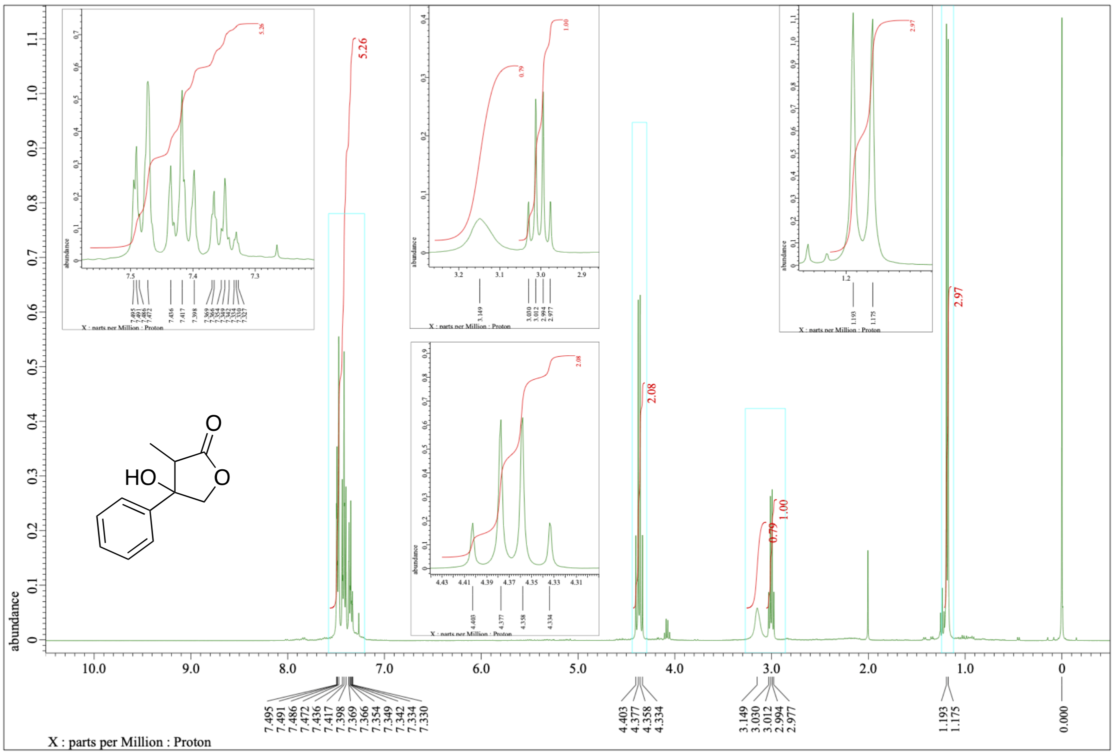

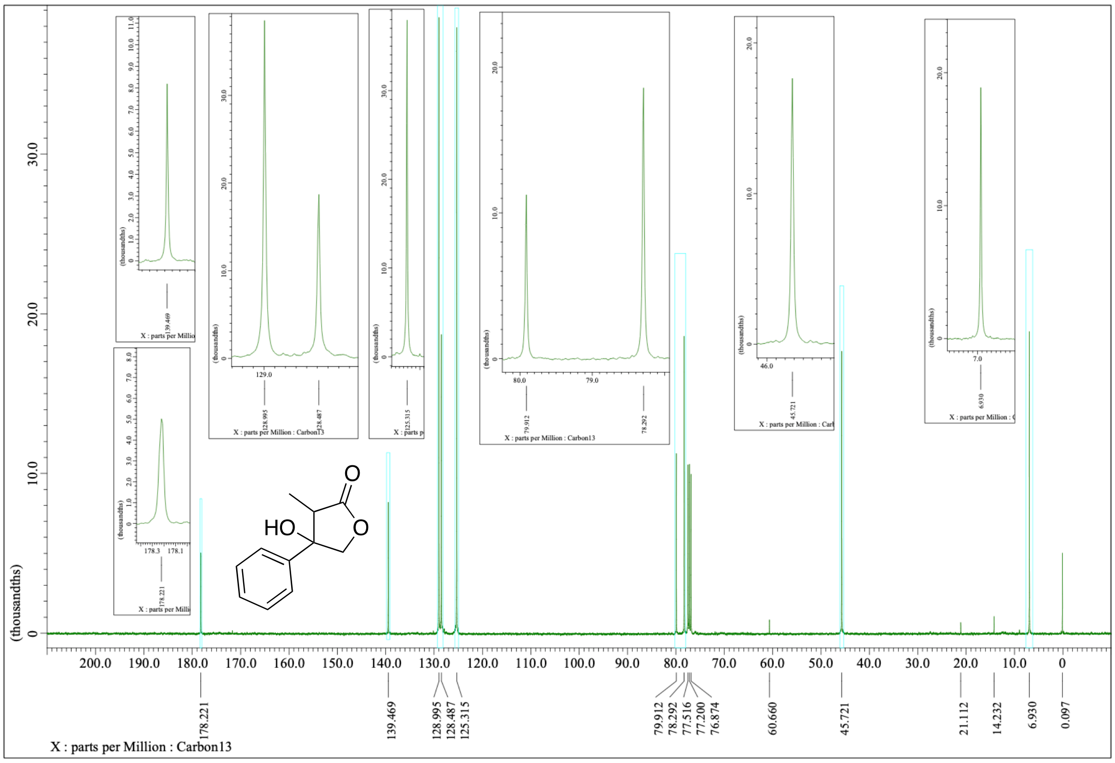

^{1}H NMR (400 MHz) and ^{13}C NMR (100 MHz) spectra of 2d ($CDCl_3$)

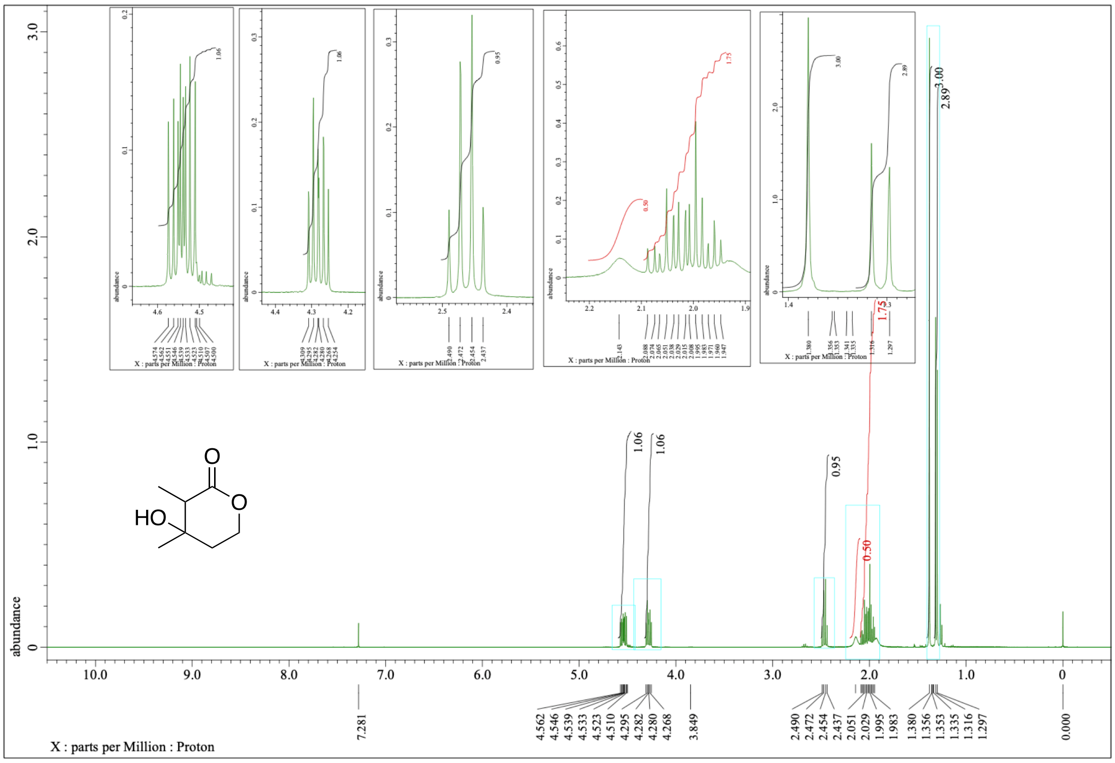

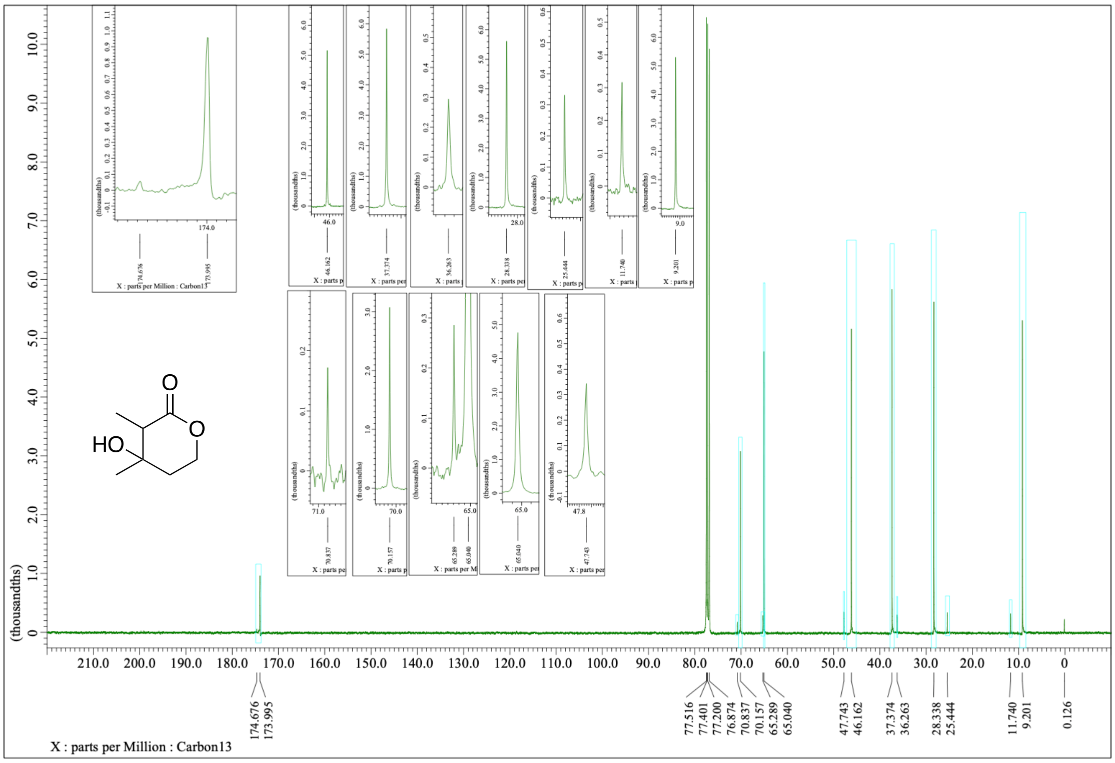

^{1}H NMR (400 MHz) and ^{13}C NMR (100 MHz) spectra of 2e ($CDCl_3$)

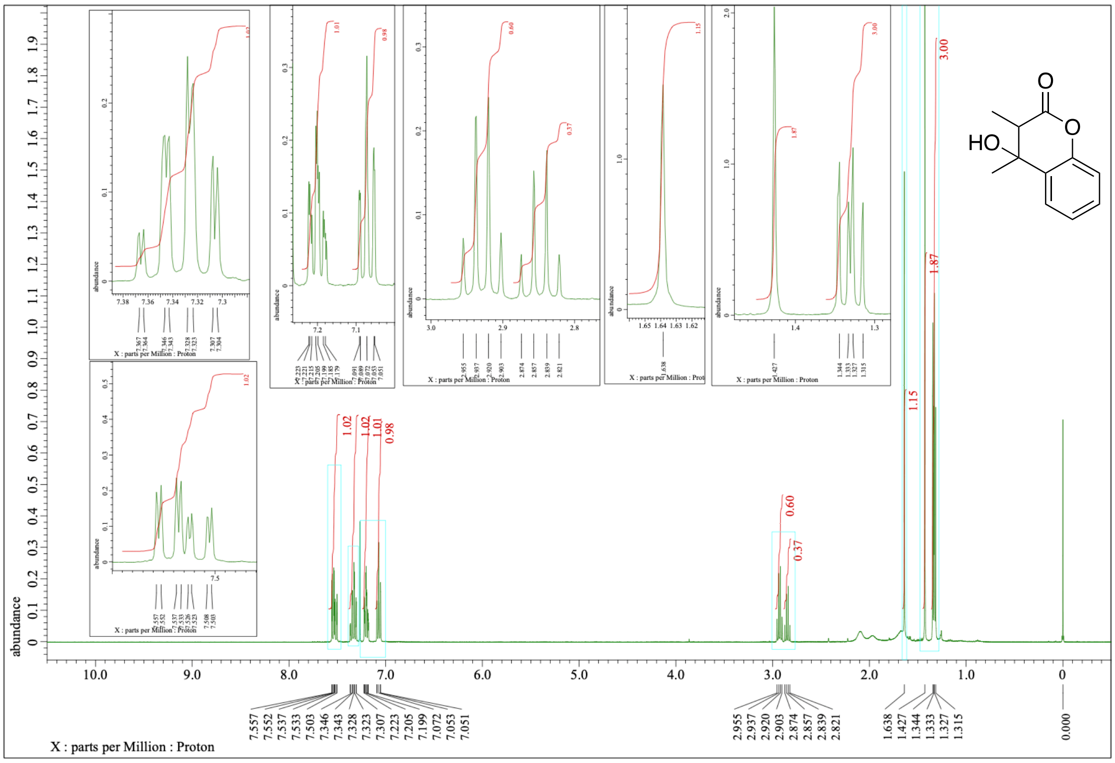

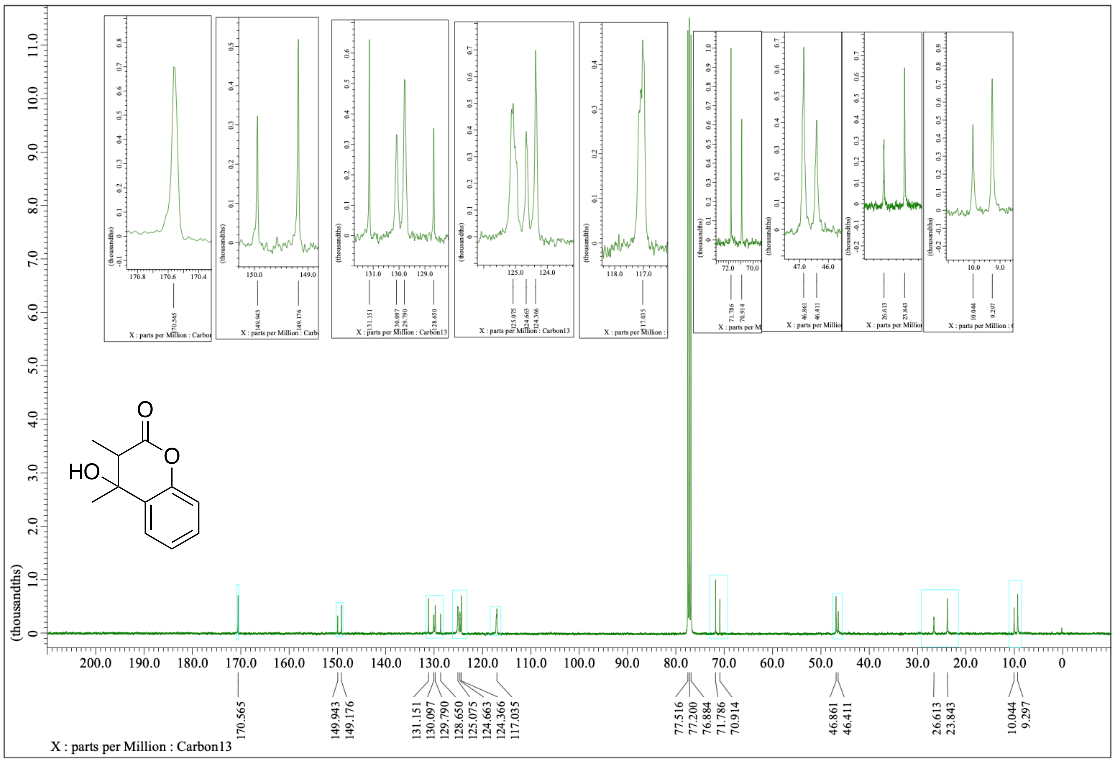

^{1}H NMR (400 MHz) and ^{13}C NMR (100 MHz) spectra of 2g ($CDCl_3$)

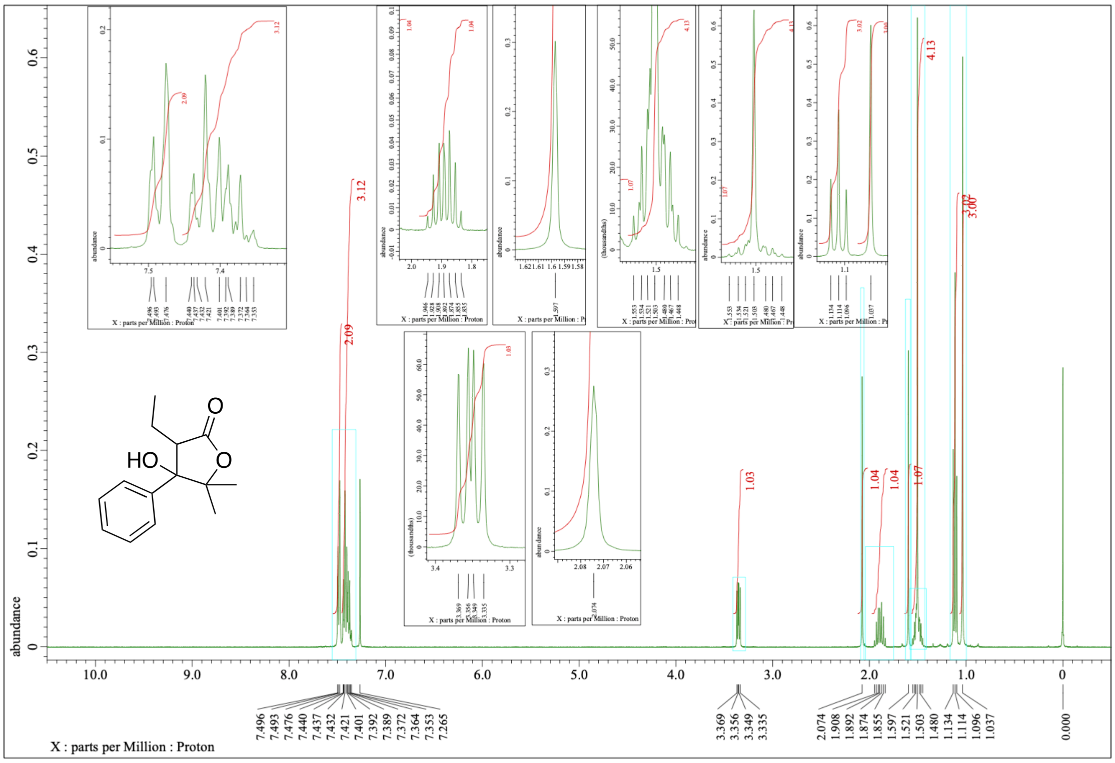

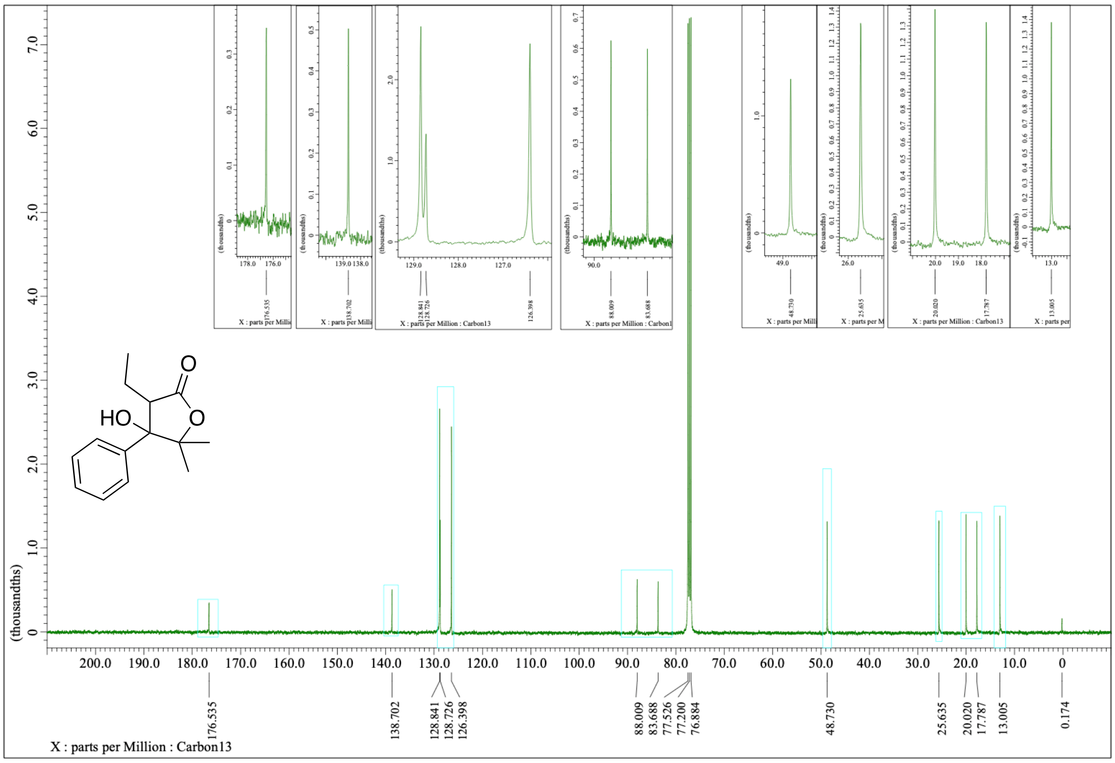

^{1}H NMR (400 MHz) and ^{13}C NMR (100 MHz) spectra of 2h (CDCl₃)

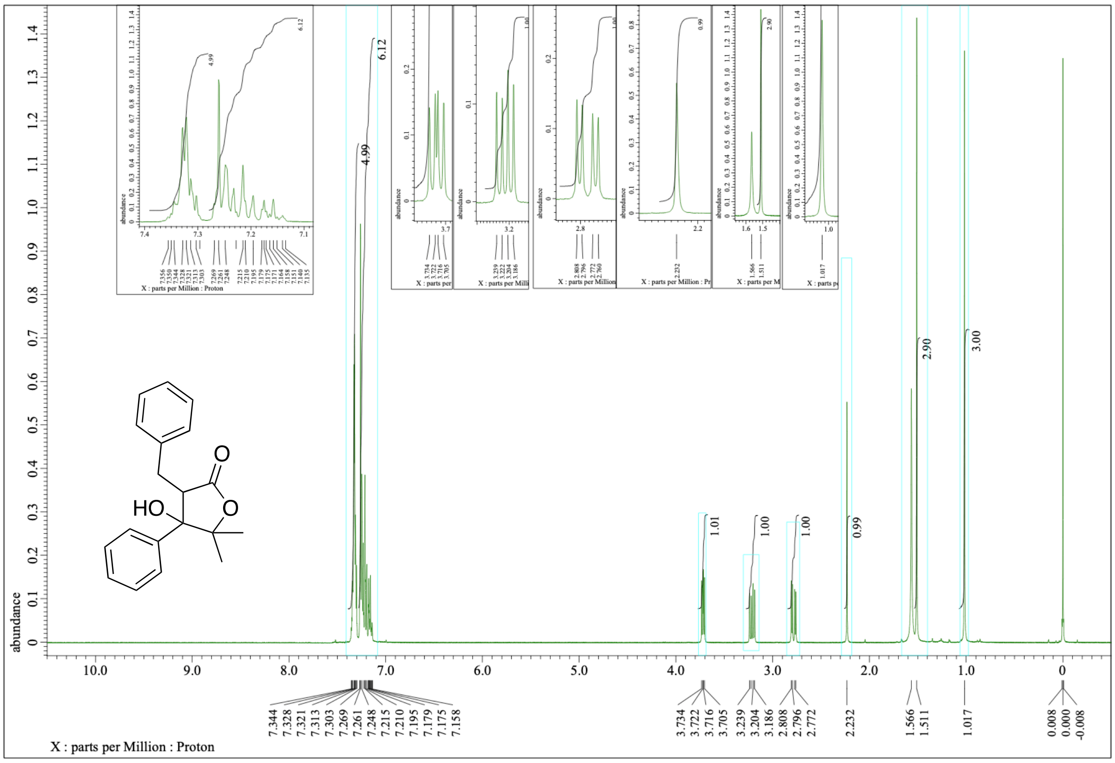

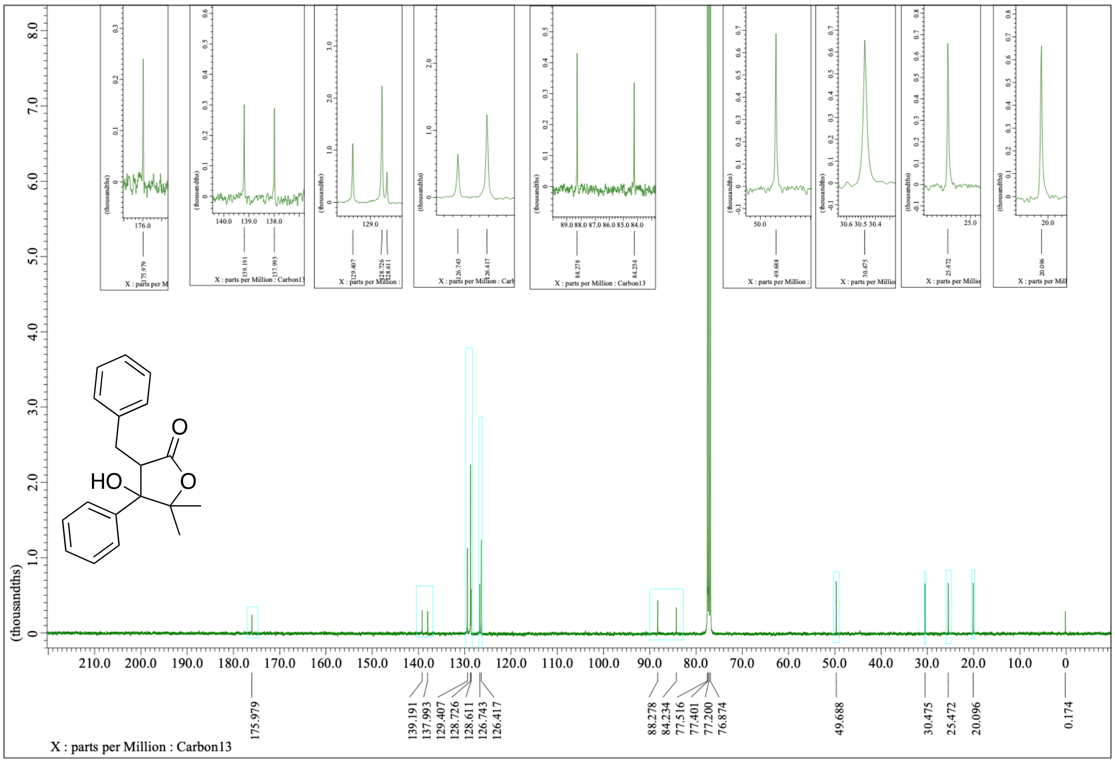

^{1}H NMR (400 MHz) and ^{13}C NMR (100 MHz) spectra of 2j ($CDCl_3$)

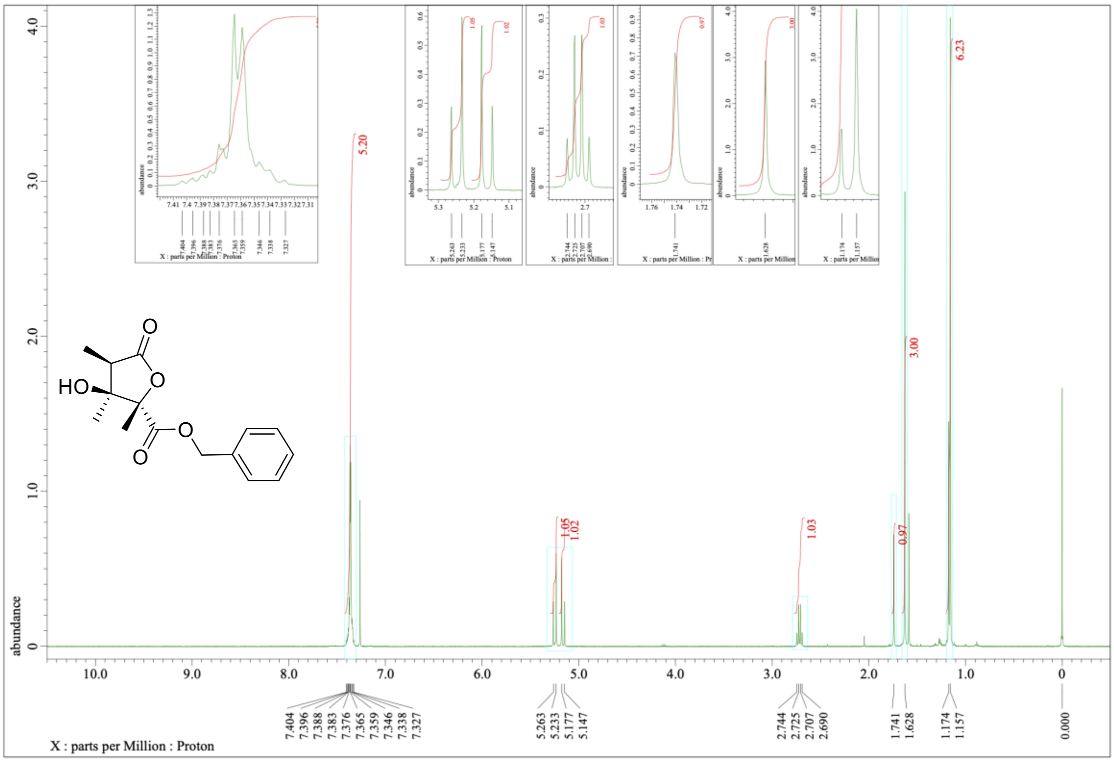

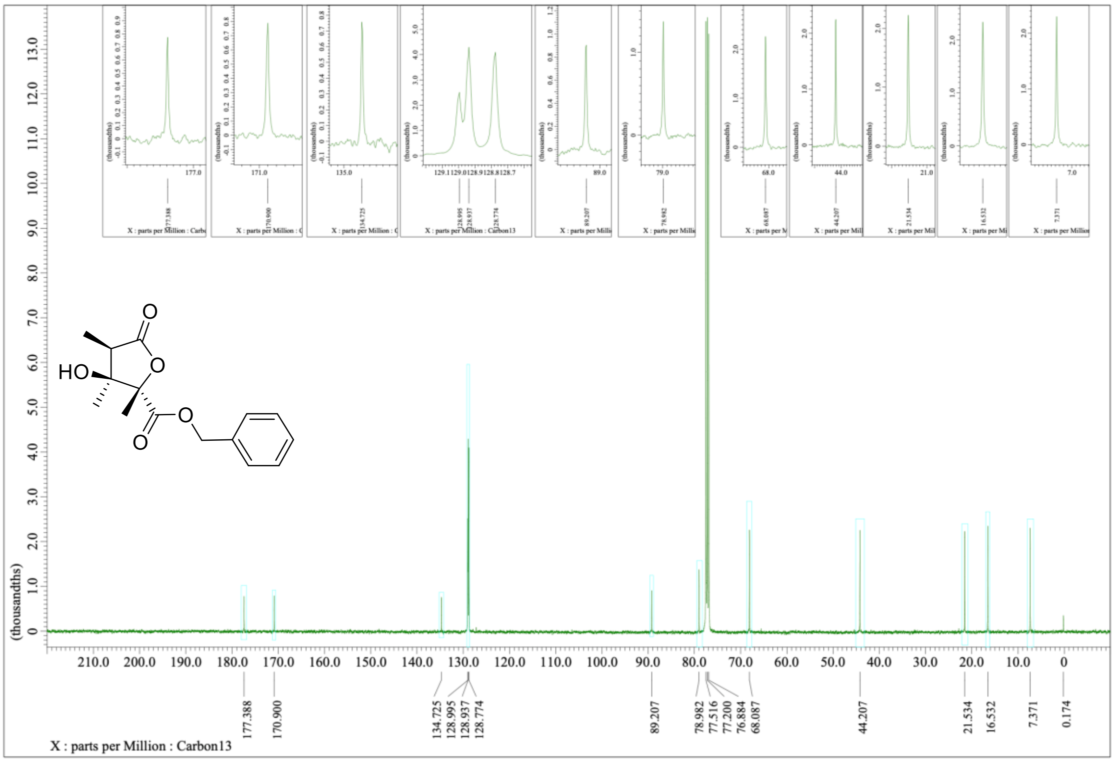

Supplement: File 1 — General procedures and analytical data, including copies of 1H NMR, 13C NMR, and X-ray crystallography. [file Beilstein_J_Org_Chem-18-1642-s001.pdf]
